# Preliminary Lectures

**Published:** 1851-10

**Authors:** 


### PRELIMINARY LECTURES.

With the issue of our present number, the October course of Lectures opens in the University of Louisville. The Professors by thi preliminary course consider that they are virtually extending the term by one month, and thus assent to the wisdom of the innovation recommended by the American Medical Association. Besides the professors, Dr. Richardson, demonstrator of Anatomy, Dr. D. W. Yandell, Dr. R. J. Breckinridge, Jr., Dr. William H. Cobb, and Dr. William Miller will participate in this course, which embraces clinical lectures and instruction in practical anatomy. We think we are safe in promising students an instructive course of lectures.

